# A Case of Hemoptysis and Menorrhagia

**Published:** 1846-05

**Authors:** D. E. Harrison

**Affiliations:** Garrard county, Ky.


### A CASE OF HEMOPTYSIS AND MENORRHAGIA SUCCESSFULLY TREATED BY TARTRATE OF IRON.

By D. E. Harrison, M.D., of Garrard county, Ky.

Mrs. King, æt. 40 years, of feeble constitution, the mother of eight children, the youngest of which being two years old, was attacked suddenly on the 16th of November last with spitting of blood, and in a few hours discharged what was supposed to amount to half a gallon. I found her on the same day pale, insensible, pulseless at the wrist, breathing quick, and with cold extremities. No abnormal sounds could be detected on percussion in any part of the lungs. Stimulants and astringents having been administered, the hemorrhage soon ceased, and the patient regained her consciousness. On inquiring into the history of her case I was informed that she had been subject to hemoptysis during the long period of fifteen years, the hemorrhage being very much increased at her catamenial terms; and I was also told that the complaint was hereditary. Her health had suffered seriously by the drain, as well, probably, as by the long-continued use of medicines prescribed for its suppression. The uterine discharge, it was stated, consisted of blood which coagulated like that from the lungs.

Having just read the lectures of Dr. Buck published in the Western Journal, I was encouraged to try chalybeates and a nourishing diet, with a view to the improvement of the quality of her blood. Accordingly I put her upon the use of tartrate of iron, in doses of 8 grains gradually increased to 12 grains, three times daily, in pills combined with a little rhubarb so as to keep the bowels soluble. The improvement in her case was speedily manifest. Her complexion became healthful; her strength increased daily; she had no return of the pulmonary hemorrhage: her menses became regular, and on the 15th of the present month I ceased to attend her, considering her cured.

March 23d, 1846.

